# IL-11 is essential in promoting osteolysis in breast cancer bone metastasis via RANKL-independent activation of osteoclastogenesis

**DOI:** 10.1038/s41419-019-1594-1

**Published:** 2019-04-30

**Authors:** Mengmeng Liang, Qinyu Ma, Ning Ding, Fei Luo, Yun Bai, Fei Kang, Xiaoshan Gong, Rui Dong, Jingjin Dai, Qijie Dai, Ce Dou, Shiwu Dong

**Affiliations:** 1 Department of Biomedical Materials Science, Third Military Medical University, Chongqing, 400038 China; 2 Department of Orthopedics, Southwest Hospital, Third Military Medical University, Chongqing, 400038 China; 3 Department of promoting osteolysisBlood Purification, General Hospital of Shenyang Military Area Command, Shenyang, 110000 China; 4 State Key Laboratory of Trauma, Burns and Combined Injury, Third Military Medical University, Chongqing, 400038 China

**Keywords:** Phosphoinositol signalling, Mechanisms of disease

## Abstract

A variety of osteolytic factors have been identified from breast cancer cells leading to osteolysis, but less is known about which factor plays an essential role in the initiation process prior to the overt vicious osteolytic cycle. Here, we present in vitro and in vivo evidences to clarify the role of interleukin-11 (IL-11) as an essential contributor to breast cancer bone metastasis mediated osteolysis. Animal studies showed that bone specific metastatic BoM-1833 cells induce earlier onset of osteolysis and faster tumor growth compared with MCF7 and parental MDA-MB-231 cells in BALB/c-nu/nu nude mice. IL-11 was further screened and identified as the indispensable factor secreted by BoM-1833 cells inducing osteoclastogenesis independently of receptor activator of nuclear factor κB ligand (RANKL). Mechanistic investigation revealed that the JAK1/STAT3 signaling pathway as a downstream effector of IL-11, STAT3 activation further induces the expression of c-Myc, a necessary factor required for osteoclastogenesis. By inhibiting STAT3 phosphorylation, AG-490 was shown effective in reducing osteolysis and tumor growth in the metastatic niche. Overall, our results revealed the essential role and the underlying molecular mechanism of IL-11 in breast cancer bone metastasis mediated osteolysis. STAT3 targeting through AG-490 is a potential therapeutic strategy for mitigating osteolysis and tumor growth of bone metastatic breast cancer.

## Introduction

Breast cancer is a common cancer in women and a major cause of cancer death due to the development of secondary tumors in vital organs^1^. While surgical therapies for primary tumors are generally effective, metastasis is often refractory. An estimating eighty percent of breast cancer patients develop bone metastasis as the disease advances. Bone metastases from breast cancer are typically osteolytic, involving the activation of osteoclasts that cause pathological bone resorption, with intense pain, bone fractures, nerve compression, and hypercalcemia^2^. The development of osteolytic lesions is based on complex interactions between cancer cells and osteoclasts in a vicious cycle of bone destruction and tumor expansion^3,4^. Breast cancer cells produce a variety of osteolytic factors leading to osteoclastic bone resorption, the subsequent release of bone matrix-embedded transforming growth factor beta (TGF-β) in turn promotes tumor growth^5,6^.

Two important regulating factors, receptor activator of nuclear factor κB ligand (RANKL) and macrophage-colony stimulating factor (M-CSF) are necessary for osteoclast differentiation and survival^7,8^. Nevertheless, osteoclastogenesis can also be stimulated non-canonically by tumor necrosis family alpha (TNFα), TGF-β, interleukin-6 (IL-6), lysyl oxidase (LOX), insulin-like growth factor-I (IGFI) and IGF-II^9–11^. Breast cancer cell is known to produce numerous osteolytic factors including parathyroid hormone-related protein (PTHrP), IL-1, IL-6, IL-8, IL-11, vascular endothelial growth factor (VEGF), connective tissue growth factor (CTGF), matrix metallopeptidase 1 (MMP1), hepatocyte growth factor, etc^12–16^. Some of these factors activate osteoclastogenesis by increasing RANKL expression from osteoblast while some activate osteoclastogenesis synergistically with RANKL stimulation or in a RANKL independent way.

In contrast to our knowledge of the abundant osteolytic factors, less is known about the initiation process prior to the overt vicious osteolytic circle. IL-11 is a member of the IL-6 family, and both cytokines share gp130 as the common signal transducer^17^. Both IL-6 and IL-11 are osteolytic factors produced by breast cancer cells and their expression is associated with the development of bone metastases^18^. Differently, IL-6 induces osteoclastogenesis dependent on osteoblastic cells while IL-11 induces osteoclast differentiation directly^19,20^. It has been shown the breast cancer cell line overexpressing IL-11 increases tumor burden and osteolytic lesions in mouse bone metastasis model^21^. In addition, human breast cancer tumors expressing IL-11 have higher rates of bone metastasis occurrences^22^. However, it still remains questionable if IL-11 induces osteoclast differentiation independent of RANKL or just enhances osteoclastogenesis synergistically with RANKL.

In this study, we present both in vivo and in vitro evidences to clarify the essential roles and the underlying molecular mechanisms of IL-11 in breast cancer bone metastasis mediated osteolysis. We identify IL-11 induces osteoclastogenesis independently of RANKL via JAK1/STAT3 activated c-myc pathway. Blocking of STAT3 phosphorylation in vivo effectively inhibits osteolysis and tumor growth of metastatic breast cancer.

## Materials and methods

### Cells and treatment

Luciferase-labeled MCF7, MDA-MB-231, and BoM-1833 cells were established from the respective cell line (American Type Culture Collection; ATCC). In Short, cells were infected with UBI-MCS-EGFP-SV40-FIREFLY-Luciferase-IRES-Puromycin and 5 μg/mL polybrene added to the cultures. After overnight culturing medium was changed, cells were split 48 h later, and grown thereafter in 4 μg/mL puromycin for selection of infected cells. si-RNA against mouse STAT3, c-Myc, and control si-RNA (Santa Cruz Biotechnology) were transfected into BMMs using Lipofectamine RNAiMax Reagent (Invitrogen). RAW264.7 cells (American Type Culture Collection, ATCC) were cultured in Dulbecco’s modified Eagle’s medium (DMEM) supplemented with 10% fetal bovine serum (FBS), 100 U/ml penicillin (Gibco), and 100 U/ml streptomycin (Gibco) in a 37 ℃ incubator containing 5% CO2-enriched atmosphere. BMMs were cultured with condition medium (CM) of different breast cancer cell lines with M-CSF (50 ng/ml) for 4 days. To block corresponding osteolytic factors in the CM of BoM-1833 cells, we used monoclonal antibodies against VEGF (Abcam), PTHrP (Abcam), IL-11 (Abcam) and CTGF (Abcam) in a concentration of 50 μg/ml, and cultured for 96 h.

### In vitro osteoclastogenesis assays and evaluations

Bone marrow macrophages (BMMs) were separated and cultured with M-CSF (50 ng/ml) for 24 h to obtain BMMs. Cells were cultured in α-minimal essential medium (MEM) containing 10% FBS and 1% Penicillin-streptomycin solution. For tartrate resistant acid phosphatase (TRAP) stain, cells were cultured in a 96-well plate at a density of 5 × 10^3^ cells/well with different stimulations. Cells were fixed in 4% paraformaldehyde for 20 min and then stained with TRAP staining solution (0.1 mg/ml of naphthol AS-MX phosphate, 0.3 mg/ml of Fast Red Violet LB stain) according to the manufacturers’ instructions. Relative TRAP activity was measured by colorimetric analysis. For immunofluorescent stain, cells were cultured on glass sheet in a 12-well plate at a density of 4 × 10^4^ cells/well with different stimulations. In brief, on day 4, cells were washed and fixed for permeabilization. After blocking, primary antibody (Anti-Vinculin) was then diluted to a working concentration (1:300) in blocking solution, and cells were incubated for 1 h at room temperature. Secondary antibody (Alexa Fluor 488 Goat Anti-Mouse IgG (H + L) Antibody, Invitrogen) (1:500) and TRITC conjugated Phalloidin (1:500) was diluted in 1 × PBS and cells were incubated for 1 h at room temperature. Nuclei counterstaining was performed by DAPI (1:1000) for 5 minutes followed by fluorescence microscopy.

### Immunohistochemistry (IHC)

Hindlimbs were removed from mice at the time of sacrifice and bones were fixed in 10% neutral-buffered formalin, washed and decalcified in a solution of 10% EDTA for 2 weeks, and embedded in paraffin. Immunohistochemical analysis was performed with heat-induced antigen retrieval in sodium-citrate buffer (Dako). Primary antibodies used was anti-phospho-STAT3 at 1:100 (Santa Cruz), anti-IL-11 at 1:200 (Santa Cruz), anti-c-Myc at 1:100 (Santa Cruz), anti-pJAK1 at 1:100 (Santa Cruz). Biotinylated secondary antibody was used with the EnVision + system HRP kit (Dako) and nuclei were counterstained with hematoxylin.

### Animal experiments

All procedures involving mice and experimental protocols were approved by Institutional Animal Care and Use Committee of Third Military Medical University. Mice for all experiments were females 6–8 weeks of age. For bone metastasis studies, 1 × 10^6^ tumor cells were resuspended in PBS and injected into the femur of anesthetized female BALB/c-nu/nu nude mice. The STAT3 inhibitor AG-490 (MCE) were dissolved in PBS and was injected intravenoso (i.v.) every two days for a continuous three weeks at a concentration of 5 mg/kg. Development of tumor was monitored every week. Bioluminescence imaging and μCT analysis were performed weekly for a continuous four weeks. After 4-week treatment, mice were killed, hindlimbs were removed and fixed in 10% neutral-buffered formalin for TRAP or immunohistochemical staining. A minimum of 8 mice were used per group.

### Bioluminescence imaging (BLI) and analysis

Anesthetized mice were intraperitoneally injected with 75 mg/kg D-Luciferin (Fanbo Biochemicals) in PBS. Bioluminescence images were acquired by using the IVIS Imaging System (Perkin Elmer) 2–5 min after injection. Acquisition times at the beginning of the time course started at 60 s and were reduced in accord with signal strength to avoid saturation. Analysis was performed with Living Image software by measuring photon flux using a region of interest drawn around the bioluminescence signal to be measured.

### μCT analysis and histological analysis

For μCT analysis, Bruker MicroCT Skyscan 1272 system (Kontich, Belgium) with an isotropic voxel size of 10.0μm was used to image the whole femur. Scans were conducted in 4% paraformaldehyde and used an x­ray tube potential of 60 kV, an x­ray intensity of 166 μA, and an exposure time of 1700 ms. For trabecular bone analysis of the distal femur, an upper 3­ mm region beginning 0.8 mm proximal to the most proximal central epiphysis of the femur was contoured. For cortical bone analysis of femur (2D analysis), a 0.5­ mm region beginning 4.5 mm proximal to the most proximal central epiphysis of the femur. Trabecular and cortical bones were thresholded at 86–255 (8 bit grey scale bitmap). 3D images were obtained from contoured 2D images by methods based on distance transformation of the grey scale original images (CTvox, Ver. 3.0.0). 3D and 2D analysis were performed using software CT Analyser (Ver. 1.15.4.0). All images presented are representative of the respective groups.

For the bone histological analysis, femurs were dissected and fixed in 4% paraformaldehyde in PBS for 48 h. Femurs were then decalcified by daily change of 15% tetrasodium EDTA for 2 weeks. Tissues were dehydrated by passage through an ethanol series, cleared twice in xylene, embedded in paraffin, and sectioned at 8 μm thickness along the coronal plate from anterior to posterior. Decalcified femoral sections were stained with TRAP.

### Murine cytokine ELISA

Quantitative levels of murine IL-11, CTGF, VEGF, PTHrP, and TGF-β1 in serum, bone marrow isolated from mice and the conditioned media of cultured cells was determined in triplicate by ELISA according to the manufacturer’s protocol (Quantikine immunoassay kit, R&D systems).

### Statistical analysis

All data are representative of at least three experiments of similar results performed in triplicate unless otherwise indicated. Data are expressed as mean ± SD. One-way ANOVA followed by Student–Newman–Keuls post hoc tests was used to determine the significance of difference between results, with *p < 0.05, **p < 0.01 being regarded as significant.

Other methods (Reagents, Co-culture assay and CM preparation, quantitative real-time PCR, Western blotting,) were described in Supplementary Materials and Methods.

## Results

### BoM-1833 cells induce earlier onset of osteolysis and faster tumor growth

**Fig. 1:**
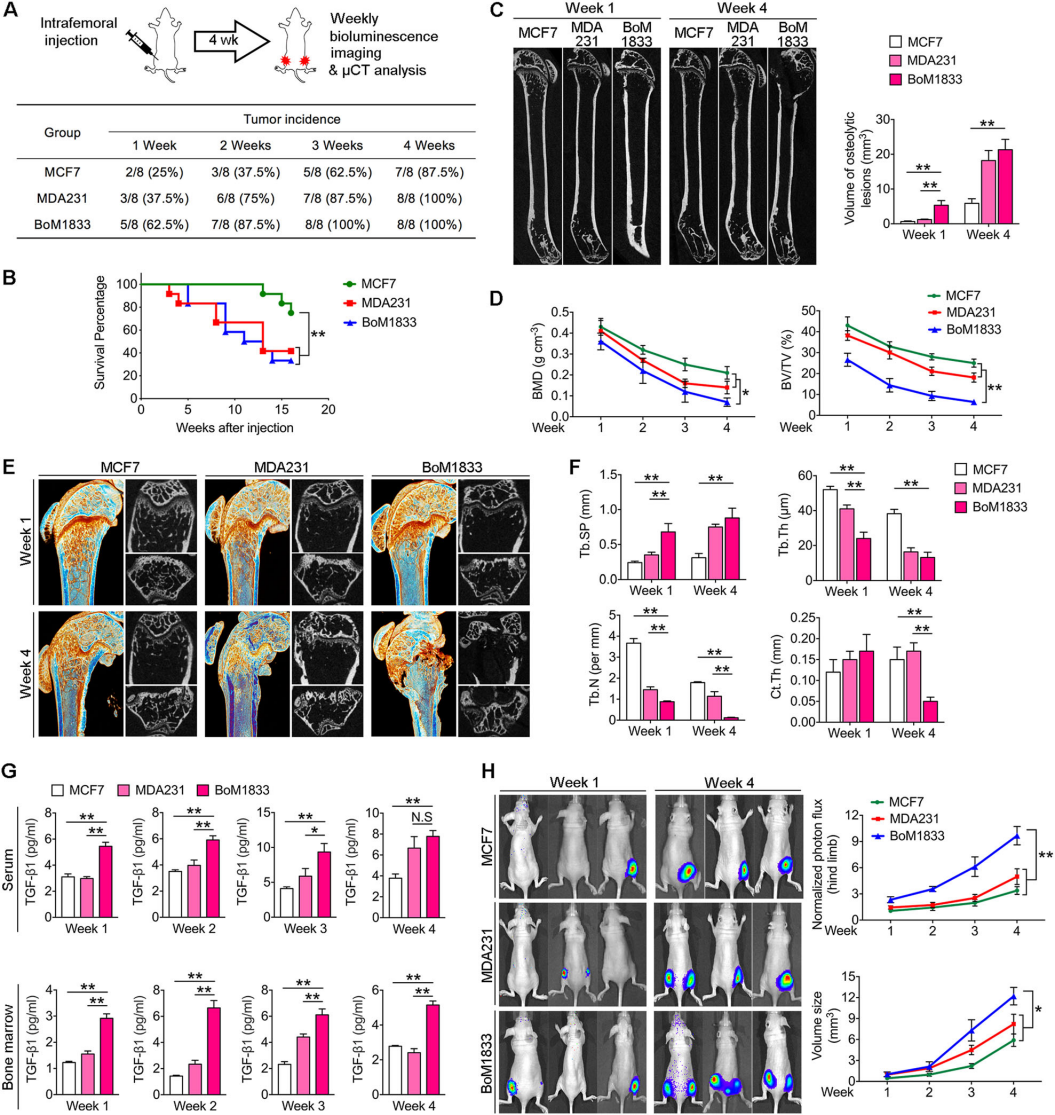
BoM-1833 cells induce earlier onset of osteolysis and faster tumor growth. **a** schematic representation of the experimental design. MCF7^Luc^, MDA-MB-231^Luc^ or BoM-1833^Luc^ cells were injected intrafemorally in female athymic mice (*n* = 8 for each group). Osteolysis and metastatic tumor growth was monitored by weekly bioluminescence imaging (BLI) and μCT for 4 weeks. Tumor incidence was shown in table below. **b** Kaplan–Meier survival analysis representing the overall survival of mice with intrafemoral injection of MCF7, MDA-MB-231 or BoM-1833 cells, **p < 0.01, log-rank test. **c** Representative μCT of longitudinal section femurs (left) and quantification of osteolytic lesion volume (right). **d** Quantification of bone mineral density (BMD) and trabecular bone volume fraction (BV/TV). **e** Representative μCT images of reconstructed distal femurs. **f** Quantification of trabecular number (Tb. N), trabecular separation (Tb. Sp), trabecular thickness (Tb.Th) and cortical thickness (Ct.Th). **g** ELISA for serum or bone marrow (BM) TGF-β concentrations. **h** Representative in vivo BLI images and quantification of normalized photon flux and tumor volume size. The data in the figures represent the averages ± SD. Significant differences are indicated as *p *<* 0.05 or **p *<* 0.01 paired using Student’s *t* test unless otherwise specified

Few studies focused on the onset and initiation of breast cancer cell induced osteolysis in bone metastases. For better observation, we intrafemorally injected female athymic mice with same amounts of MCF-7, parental MDA-MB-231 and bone metastatic BoM-1833 cells respectively and performed weekly BLI and μCT analysis for a continuous four weeks (Fig. 1a). We found that the tumorigenicity of BoM-1833 cells in week 1 (5/8) is the highest among all groups while the tumor incidence of BoM-1833 cells (8/8) is the same with parental MDA-MB-231 cells (8/8) in week 4 (Fig. 1a). Survival analysis revealed similar results that BoM-1833 and MDA-MB-231 groups showed poorer survival compared with MCF-7 groups (Fig. 1b). μCT results further revealed that BoM-1833 cells significantly increased osteolytic bone lesion volume in week 1 compared with the MCF7 and MDA-MB-231 groups, while the significance between BoM-1833 and MDA-MB-231 groups vanished in week 4 (Fig. 1c). In addition, quantification analysis showed that the bone mineral density (BMD) and trabecular bone volume fraction (BV/TV) decreased significantly in BoM-1833 group compared with the MCF7 and MDA-MB-231 groups (Fig. 1d). For trabecular and cortical bone analysis of the distal femur, an upper 3 ­mm region beginning 0.8 mm proximal to the most proximal central epiphysis of the femur was contoured as region of interest (Fig. 1e). Quantification analysis showed that trabecular separation (Tb. Sp) was increased and trabecular thickness (Tb. Th) was decreased significantly in BoM-1833 groups compared with the MCF7 and MDA-MB-231 groups in week 1, while the significance between BoM-1833 and MDA-MB-231 groups vanished in week 4. Trabecular number (Tb. N) showed significant decrease in MDA-MB-231 and BoM-1833 groups compared with MCF-7 groups both in week 1 and week 4. Additionally, cortical bone thickness (Ct. Th) was only significantly decreased by BoM-1833 cell injection in week 4 (Fig. 1f). To verify whether the increased bone resorption by BoM-1833 cell injection is associated with elevated TGF-β release, we then used ELISA to detect the TGF-β levels in serum and bome marrow. The results showed that serum TGF-β level is significantly higher in BoM-1833 groups compared with the MCF7 and MDA-MB-231 groups from week 1 to 3, while the significance between BoM-1833 and MDA-MB-231 groups vanished in week 4. The bone marrow TGF-β level of BoM-1833 group is significantly higher compared with the MCF7 and MDA-MB-231 groups throughout the four weeks (Fig. 1g). BLI results showed that BoM-1833 cell injection significantly increased hindlimb tumor burden compared with MCF7 and MDA-MB-231 cell injections (Fig. 1h). These data suggest that bone specific metastatic BoM-1833 cell compared with MCF7 and parental MDA-MB-231 cell showed earlier onset of osteolysis and faster tumor growth due to the increased release of TGF-β.

### IL-11 is indispensable in BoM-1833 condition medium activated osteoclastogenesis

**Fig. 2:**
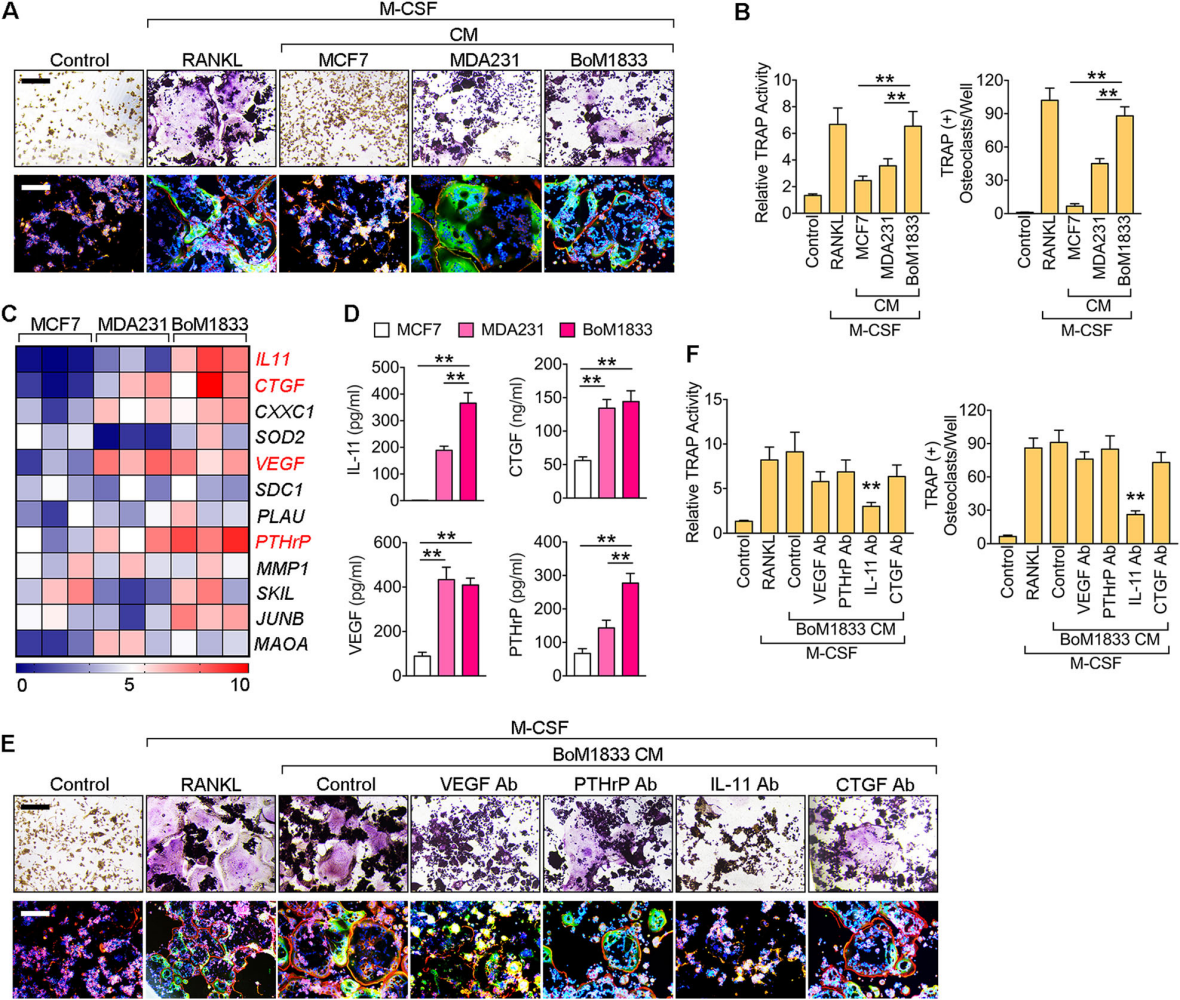
IL-11 is indispensable in BoM-1833 condition medium activated osteoclastogenesis. **a** Representative TRAP stain and immunostaining images (F-actin: green; Phalloidin: red) of bone marrow macrophages (BMMs) treated with PBS (control), M-CSF + RANKL, and M-CSF + condition medium (CM) from MCF-7, MDA-MB-231 or BoM-1833 cells. Bar represents 200 μm. **b** Quantification of relative TRAP activity and osteoclast number per well in (**a**). **c** Profiling of differentially expressed genes between MCF-7, MDA-MB-231 and BoM-1833 cells. Genes highlighted in red were further functionally tested. **d** ELISA for IL-11, CTGF, VEGF and PTHrP concentrations from MCF-7, MDA-MB-231 and BoM-1833 cells. **e** Representative TRAP stain and immunostaining images (F-actin: green; Phalloidin: red) of BMMs treated with PBS (control), M-CSF + RANKL, M-CSF + BoM-1833 CM, and M-CSF + BoM-1833 CM with monoclonal antibodies against VEGF, PTHrP, IL-11 or CTGF. Bar represents 200 μm. **f** Quantification of relative TRAP activity and osteoclast number per well in (**e**). Significance mark above IL-11 Ab group was acquired by comparisons among positive control and all Ab treated groups. The data in the figures represent the averages ± SD. Significant differences are indicated as *p *<* 0.05 or **p *<* 0.01 paired using Student’s *t* test unless otherwise specified

To investigate the in vitro effects of different breast cancer cell lines in osteoclastogenesis, we first co-cultured different breast cancer cell lines with preosteoclasts (Supplementary Fig. S1a). TRAP stain results suggested that preosteoclasts co-cultured with BoM-1833 cells showed significantly higher TRAP activity and more osteoclast number (Supplementary Fig. S1b). BMMs cultured with condition medium (CM) of different breast cancer cell lines showed similar results that BoM-1833 CM induces the highest level of osteoclastogenesis characterized by the highest TRAP activity and the most osteoclast number independent of RANKL (Fig. 2a, b). Microarray profiling revealed differentially expressed genes between MCF-7, MDA-MB-231 and BoM-1833 cells and four osteolysis related genes, namely *IL-11*, *CTGF*, *VEGF* and *PTHrP* were screened with highest expression in BoM-1833 cells (Fig. 2c). ELISA results then validated that IL-11 and PTHrP releases were the highest in BoM-1833 cells. To determine the most essential osteolytic factor released by BoM-1833 cells, we used monoclonal antibodies against VEGF, PTHrP, IL-11 and CTGF to block corresponding osteolytic factors in the CM of BoM-1833 cells in osteoclastogenesis assays (Fig. 2e). TRAP stain results revealed that osteoclastogenesis was significantly reduced by blocking of IL-11 (Fig. 2f). These data suggested that BoM-1833 CM induces osteoclastogenesis independent of RANKL via IL-11.

### IL-11 activates osteoclastogenesis independent of RANKL via JAK1/STAT3 pathway

**Fig. 3:**
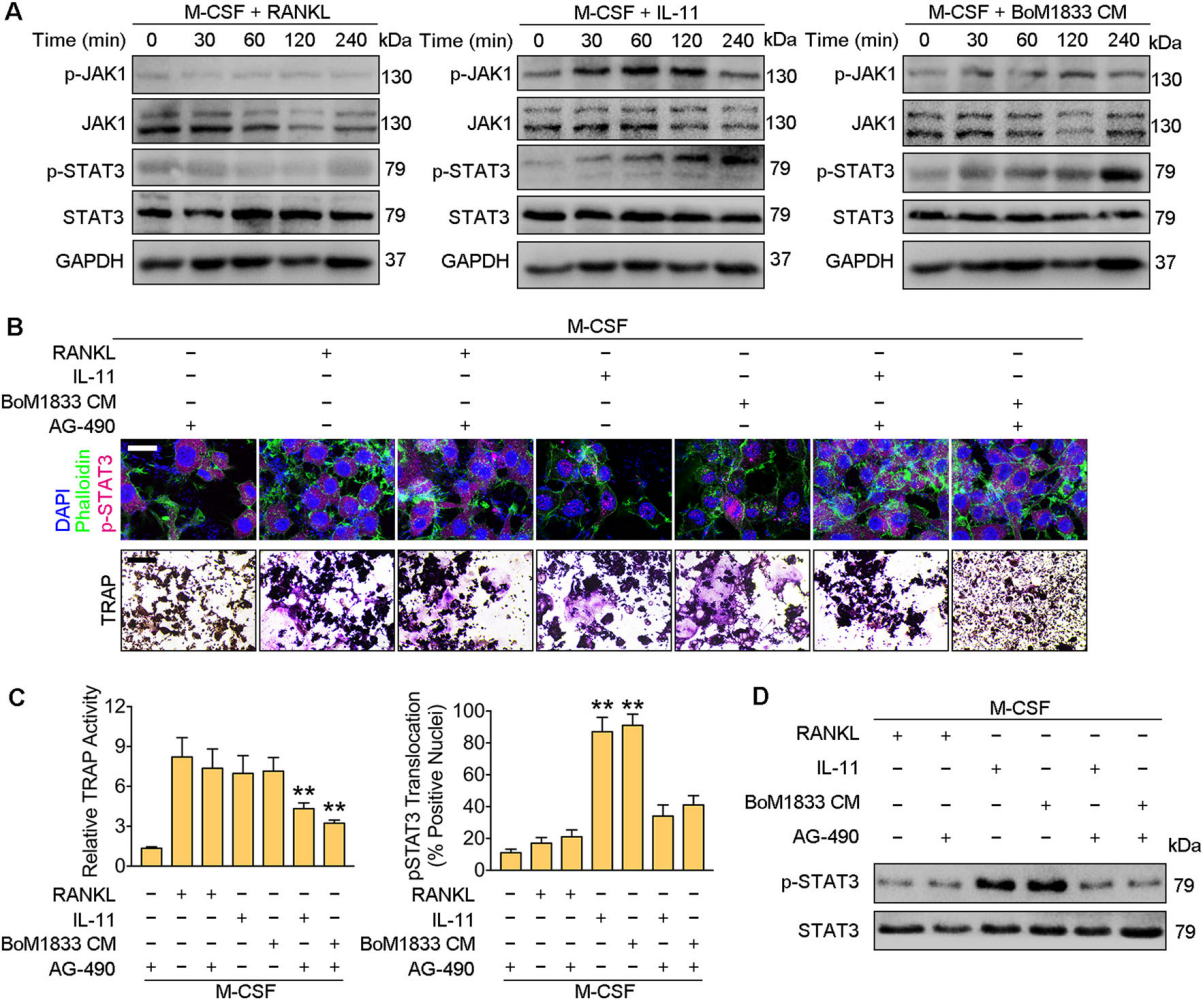
IL-11 activates osteoclastogenesis independent of RANKL via JAK1/STAT3 pathway. **a** Western blot analysis of p-JAK1, JAK1, p-STAT3, STAT3 and GAPDH in BMMs treated with M-CSF + RANKL, M-CSF + IL-11 or M-CSF + BoM1833 CM. **b** Immunostaining of phalloidin (green) and p-STAT3 (violet) and TRAP stain images of BMMs induced with M-CSF + RANKL, M-CSF + IL-11 or M-CSF + BoM1833 CM with or without AG-490. Bar represents 20 μm for Immunostaining and 200 μm for TRAP stain. **c** Quantification of relative TRAP activity and p-STAT3 positive nuclei number in (**b**). **d** Western blot analysis of p-STAT3 and STAT3 in BMMs induced with M-CSF + RANKL, M-CSF + IL-11 or M-CSF + BoM1833 CM with or without AG-490. The data in the figures represent the averages ± SD. Significant differences are indicated as *p *<* 0.05 or **p *<* 0.01 paired using Student’s *t* test unless otherwise specified

To understand the underlying mechanism in IL-11 activated osteoclastogenesis, we detected the juxta-positioning of the intracellular Janus-1 (JAK1)/signal-transducer and activated of transcription-3 (STAT3) pathway downstream of IL-11/IL-11Rα interaction^23,24^. The activation JAK1/STAT3 pathway was analyzed in osteoclastogenesis induced by RANKL, IL-11 and BoM-1833 CM respectively. Western blot results revealed that RANKL induced osteoclastogenesis is independent of JAK1/STAT3 pathway, while both IL-11 and BoM-1833 CM induced osteoclastogenesis is dependent on the activation of JAK1 and downstream STAT3 characterized by the highest phosphorylation level of JAK1 at 120 min and the highest phosphorylation level of STAT3 at 240 min upon stimulation (Fig. 3a). We then used the JAK inhibitor AG490 to block the constitutive activation of STAT3^25^. Immunofluorescent staining of p-STAT3 results showed that IL-11 and BoM-1833 CM but not RANKL increased p-STAT3 nuclear accumulation. The results also showed that nuclear translocation of p-STAT3 induced by IL-11 and BoM-1833 was decreased by prior exposure to AG-490 (Fig. 3b). Accordingly, TRAP stain quantification showed that osteoclastogenesis induced by IL-11 and BoM-1833 but not RANKL was significantly reduced by AG-490 (Fig. 3c). We further performed western blot assays and confirmed that STAT3 activation at 240 min upon stimulation of IL-11 and BoM-1833 CM was robustly inhibited by AG-490 exposure (Fig. 3d). These data suggested that JAK1/STAT3 activation is required for IL-11 induced osteoclastogenesis but not for RANKL induced osteoclastogenesis.

### STAT3 mediated c-Myc expression is required for IL-11 induced osteoclastogenesis

**Fig. 4:**
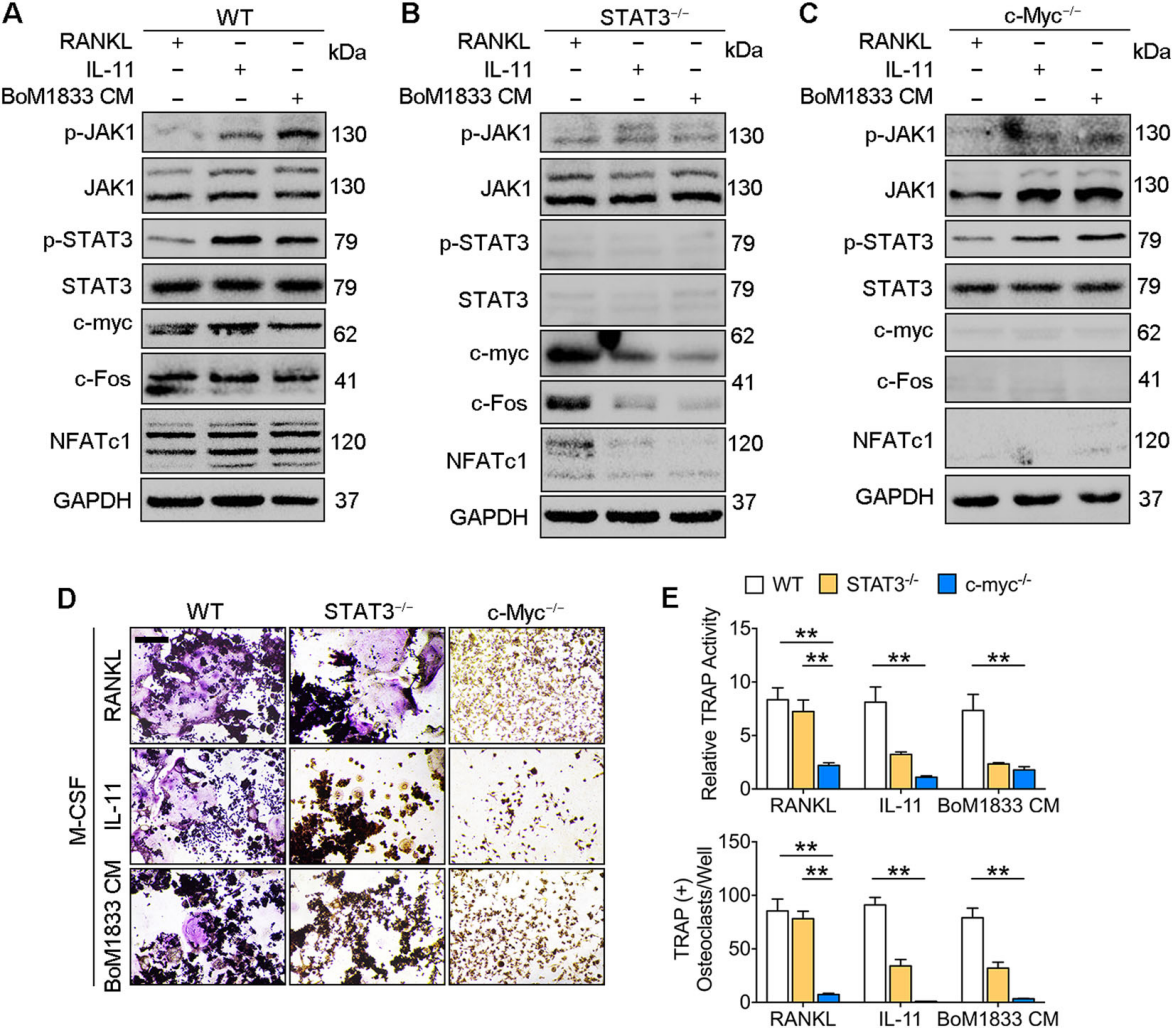
STAT3 mediated c-Myc expression is required for IL-11 induced osteoclastogenesis. **a–c** Western blot analysis of p-JAK1, JAK1, p-STAT3, STAT3, c-Myc, c-Fos, NFATc1 and GAPDH in wild type (**a**), STAT3^−/−^ **(b**) and c-Myc^−/−^ (**c**) mouse BMMs induced with M-CSF + RANKL, M-CSF + IL-11 or M-CSF + BoM1833 CM. **d** Representative TRAP stain images of wild type, STAT3^−/−^ and c-Myc^−/−^ mouse BMMs induced with M-CSF + RANKL, M-CSF + IL-11 or M-CSF + BoM1833 CM. Bar represents 200 μm. **e** Quantification of relative TRAP activity and osteoclast number per well in (**d**). The data in the figures represent the averages ± SD. Significant differences are indicated as *p *<* 0.05 or **p *<* 0.01 paired using Student’s *t* test unless otherwise specified

To explain the difference between RANKL and IL-11 induced osteoclastogenesis, we focused on the downstream molecules of STAT3 activation. We noticed that c-Myc is a downstream effector of STAT3 signaling^26^. In addition, c-Myc-dependent oxidative metabolism is required for osteoclast differentiation^27,28^. Western blot analysis revealed that IL-11 and BoM-1833 CM activated STAT3 induced c-Myc expression alike RANKL stimulation and further increased the expressions of c-Fos and nuclear factor of activated T cells 1 (NFATc1), the master regulator of osteoclastogenesis (Fig. 4a). In STAT3 deficient BMMs, both IL-11 and BoM-1833 CM failed to induce c-Myc and the downstream c-Fos, NFATc1 expressions due to the deprivation of STAT3 activation. However, RANKL stimulation of c-Myc, c-Fos and NFATc1 was not affected by STAT3 deletion (Fig. 4b). We further knockout c-Myc in BMMs and found that RANKL, IL-11 and BoM-1833 failed to activate the expressions of c-Fos and NFATc1 (Fig. 4c). TRAP stain further confirmed that STAT3 deficiency in BMMs abolished the osteoclastogenic effects of IL-11 and BoM-1833 CM while c-Myc deficiency in BMMs abolished the osteoclastogenic effects of RANKL, IL-11 and BoM-1833 CM (Fig. 4d, e). These results suggested that RANKL independent activation of osteoclastogenesis by IL-11 is realized by STAT3 mediated c-Myc expression.

### Blocking of STAT3 phosphorylation by AG-490 reduces osteolysis and tumor growth in bone metastatic breast cancer

**Fig. 5:**
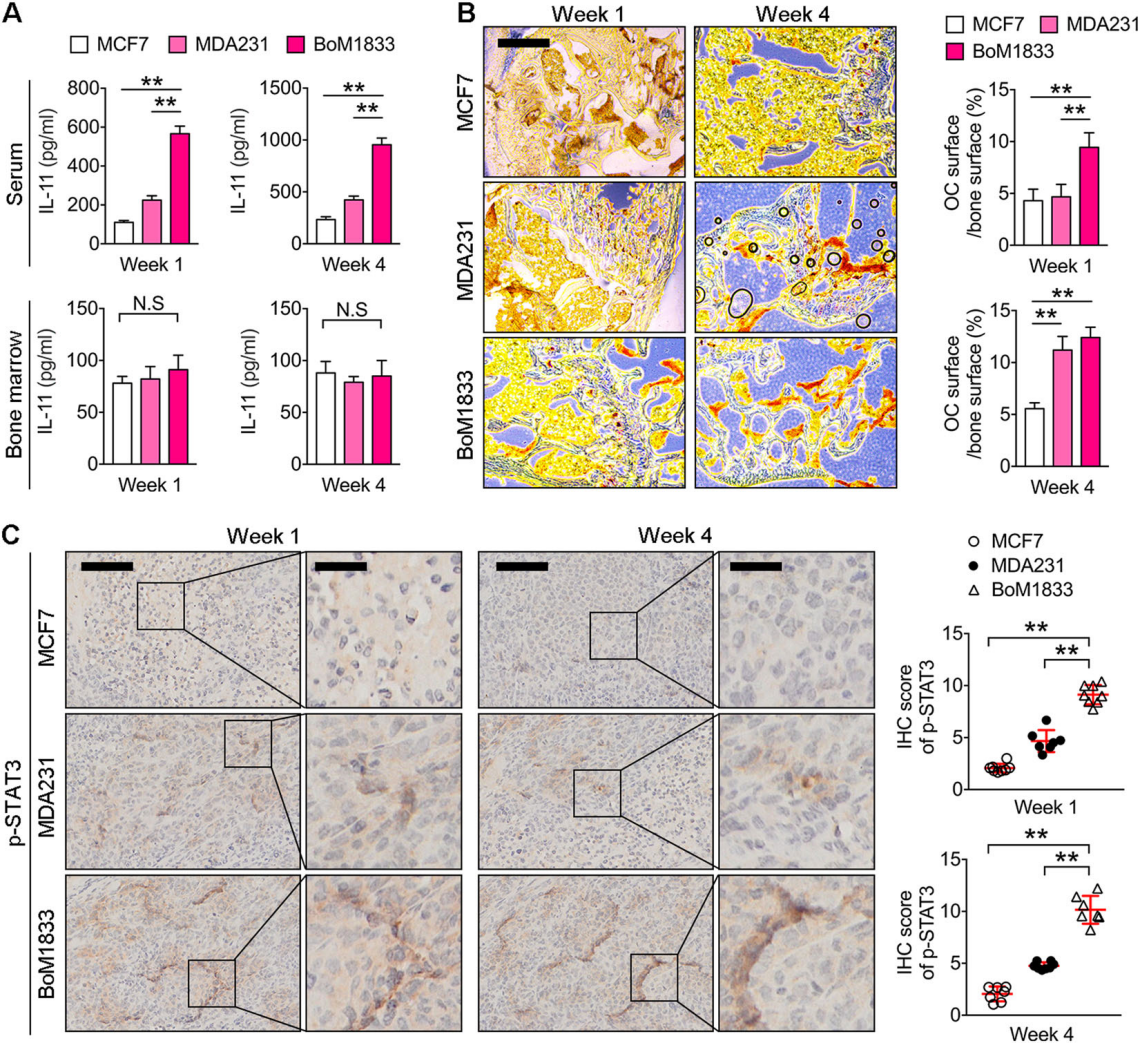
IL-11 and p-STAT3 detection in mice with intrafemoral injection of MCF7, MDA-MB-231 or BoM-1833 cells. **a** and **b** ELISA for serum and bone marrow IL-11 concentrations (**a**) and representative TRAP stain images of distal femurs (**b**) in week 1 and week 4 of mice with intrafemoral injection of MCF7, MDA-MB-231 or BoM-1833 cells. Bar represents 200 μm. Quantifications of osteoclast surface/bone surface were shown on the right. **c** Representative immunohistochemical (IHC) stain images of p-STAT3 of distal femurs in week 1 and week 4 of mice with intrafemoral injection of MCF7, MDA-MB-231 or BoM-1833 cells. Bar represents 200 μm (left) and 50 μm (right). IHC scores of p-STAT3 were quantified on the right. The data in the figures represent the averages ± SD. Significant differences are indicated as *p *<* 0.05 or **p *<* 0.01 paired using Student’s *t* test unless otherwise specified

The in vivo IL-11 level in serum was confirmed the highest in BoM-1833 groups in week 1 and week 4 by ELISA, we also found that the IL-11 level in bone marrow showed no obvious change during the time (Fig. 5a). TRAP stain results of distal femur sections also confirmed that BoM-1833 groups showed the highest in vivo osteoclastogenesis in week 1, while no significant difference was characterized between BoM-1833 and MDA-MB-231 groups in week 4 (Fig. 5b). In addition, p-STAT3 IHC results of the femur sections revealed that BoM-1833 groups showed higher p-STAT3 positive cell percentage characterized by the highest IHC score in week 1 and week 4 (Fig. 5c). Consistently, IHC results of the tumor tissue in bone showed that the expressions of IL-11, p-JAK1 and c-myc showed similar pattern compare with p-STAT3 (Supplementary Fig. S2–S4)

**Fig. 6:**
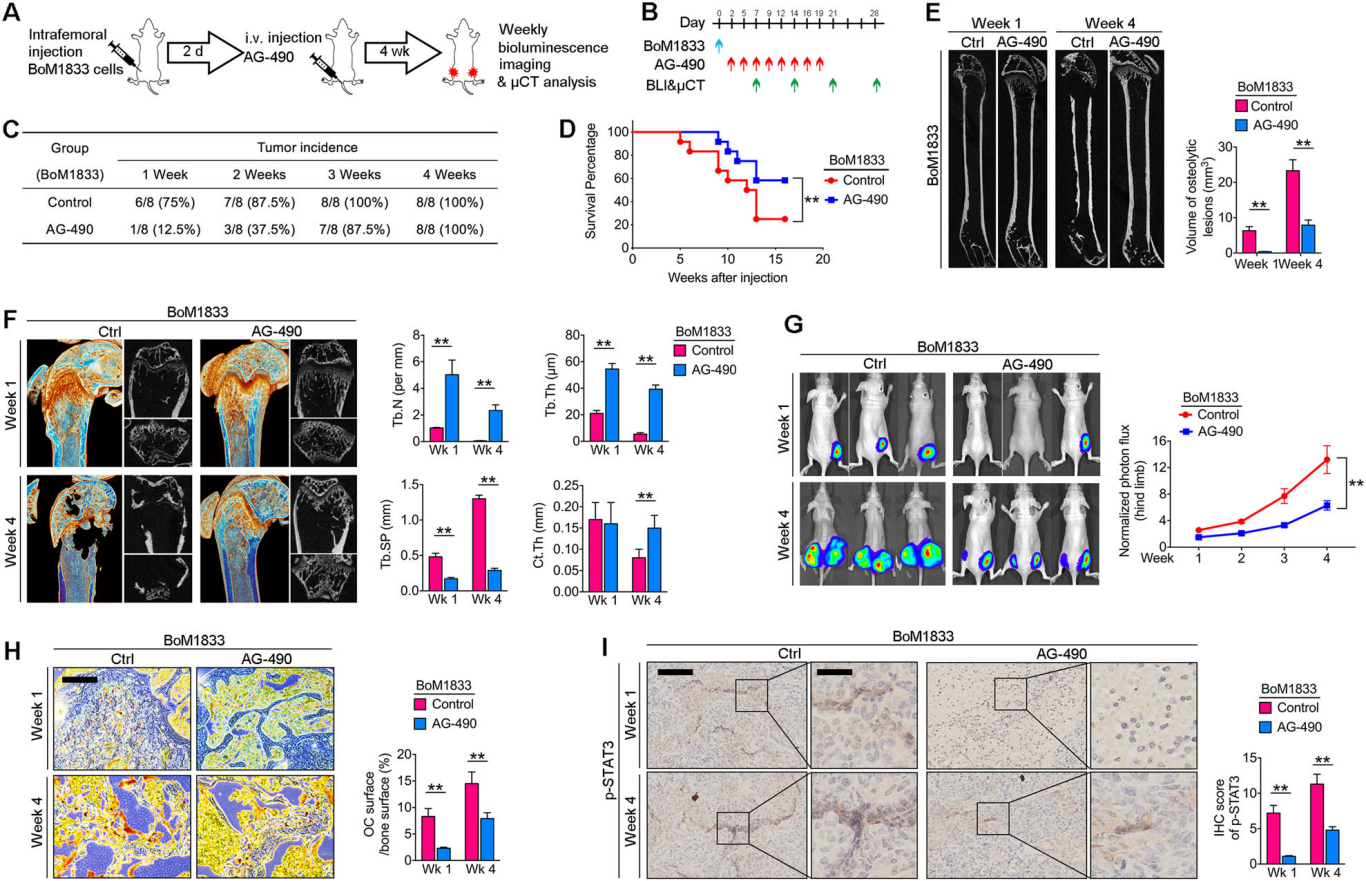
Blocking of STAT3 phosphorylation by AG-490 reduces osteolysis and tumor growth in bone metastatic breast cancer. **a** BoM-1833^Luc^ cells were injected intrafemorally in female athymic mice followed by i.v. injection of AG-490 every two days for 4 weeks (*n* = 8 for each group). **b** schematic representation of the experimental design. **c** Tumor incidence in (**a**). **d** Kaplan–Meier survival analysis representing the overall survival of BoM-1833 cell injected mice treated with normal saline (control) or AG-490, **p < 0.01, log-rank test. **e** Representative μCT of longitudinal section femurs (left) and quantification of osteolytic lesion volume (right). **f** Representative μCT images of reconstructed distal femurs. Quantification of trabecular number (Tb. N), trabecular separation (Tb. Sp), trabecular thickness (Tb.Th) and cortical thickness (Ct.Th). **g** Representative in vivo BLI images and quantification of normalized photon flux. **h** Representative TRAP stain images of distal femurs and quantification of osteoclast surface/bone surface. Bar represents 200 μm. **i** Representative immunohistochemical (IHC) stain images of p-STAT3 of distal femurs and quantification of IHC scores. Bar represents 200 μm (left) and 50 μm (right). The data in the figures represent the averages ± SD. Significant differences are indicated as *p *<* 0.05 or **p *<* 0.01 paired using Student’s *t* test unless otherwise specified

To test if blocking of STAT3 phosphorylation by AG-490 can reduce osteolysis and tumor growth in BoM-1833 injected mice, we intrafemorally injected female athymic mice with BoM-1833 cells followed by *i.v*. injection of AG-490 (5 mg/kg) every two days till week 3. BLI and μCT analysis were performed weekly for a continuous 4 weeks (Fig. 6a, b). We found that the tumorigenicity of BoM-1833 injectecd mice was significantly reduced by AG-490 treatment in week 1 and week 4 (Fig. 6c). The survival percentage was also significantly increased by AG-490 in BoM-1833 injected mice (Fig. 6d). μCT results further revealed that osteolytic bone lesion volume induced by BoM-1833 cell injection was significantly reduced by AG-490 treatment in week 1 and week 4 (Fig. 6e). The parameters of BMD and BV/TV showed similar change by AG-490 treatment (Supplementary Fig. S5). Trabecular and cortical analysis showed that AG-490 robustly restored both trabecular and cortical bone volume in BoM-1833 cell injected mice characterized by increased Tb.N, Tb. Th, Ct.Th and decreased Tb. Sp (Fig. 6f). Accordingly, the TGF-β release tested by ELISA in both serum and bone marrow showed a significant decrease in AG-490 treated groups (Supplementary Fig. S6). BLI results showed that the hindlimb tumor burden caused by BoM-1833 cell injection was significantly reduced by AG-490 treatment (Fig. 6g, Supplementary Fig. S7). TRAP stain results of distal femur sections showed that the level of in vivo osetoclastogenesis in BoM-1833 cell injected mice was reduced by AG-490 treatment in week 1 and week 4 (Fig. 6h). In addition, p-STAT3 IHC results revealed that the IHC score of p-STAT3 in BoM-1833 groups was significantly reduced by AG-490 treatment in week 1 and week 4 (Fig. 6i). Taken together, we concluded that blocking of STAT3 phosphorylation by AG-490 reduces osteolysis and tumor growth caused by BoM-1833 cell injection in athymic mice.

## Discussion

In contrast to our knowledge of overt bone metastases, it remains elusive how breast cancer cells interact with the niche cells and initiate the vicious osteolytic cycle. A previous study showed that IL-8 derived from MDA-MET cell line stimulates osteolysis independent of RANKL, whereas the detailed molecular mechanism was not clarified^29^. However, in the bone specific metastatic breast cancer cell line derived from parental MDA-MB-231 cell line characterized by Kang and colleagues, IL-11 instead of IL-8 is among the most abundantly expressed osteolytic factors^21^. Although it is well recognized that IL-11 promotes osteoclastogenesis, it remains controversial if IL-11 can directly induce osteoclastogenesis independent of RANKL. While evidence showed that IL-6 and IL-11 support osteoclast formation independent of RANKL, some argued that IL-11 can only synergistically promote RANKL induced osteoclastogenesis^30^. However, the detailed mechanism of IL-11 induced osteoclastogenesis is poorly understood. Our data indicated that IL-11 is capable of inducing osteoclastogenesis independent of RANKL by activating JAK1/STAT3 signaling pathway. We also showed that JAK1/STAT3 activation is not involved in RANKL induced osteoclast differentiation. This difference of JAK1/STAT3 activation in IL-11 and RANKL induced osteoclastogenesis can also be implied from previous findings. A study showed that STAT3 activation restrains RANKL mediated osteoclast formation suppressing expression of the E2 ubiquitin-conjugating enzyme Ubc13^31^. The same group also reported that STAT3 negatively regulates RANK signaling by inhibiting TRAF6 ubiquitination and activation of the downstream signaling^32^. Differently, a study showed that heparin acts synergistically with IL-11 to induce STAT3 activation and in vitro osteoclast formation^33^. Although JAK1/STAT3 signals exhibited converse effects in RANKL and IL-11 induced osteoclast differentiation, our results showed that both RANKL and IL-11 require c-Myc expression in activating osteoclastogenesis. Previous evidence showed that the proto-oncogene c-Myc is strongly up-regulated in mature osteoclast but not in undifferentiated cells^27^. Recently, it is reported that c-Myc transcriptional induces estrogen receptor–related receptor α (ERRα) that cooperates with NFATc1 to drive osteoclastogenesis^28^. Similar with our findings, studies also reported that c-Myc can be activated by STAT3 under different situations^26,34^. Our data confirmed that STAT3 phosphorylation induced by IL-11 further activated c-Myc expression and the downstream c-Fos and NFATc1.

In our results, we showed that IL-11 is the only osteolytic factor secreted by BoM-1833 cells that can induce osteoclast formation independent of RANKL. After screening the most enriched osteolytic factors secreted by BoM-1833 cells, only the monoclonal antibody against IL-11 successfully blocked the osteoclastogenic effect of BoM-1833 CM. We further proved that IL-11/JAK1/STAT3 induced osteoclastogenesis plays a crucial role in BoM-1833 cell induced vicious osteolytic cycle. By inhibiting STAT3 activation using AG-490, both osteolysis and tumor growth of breast cancer were strongly alleviated. A recent cohort study of 89 patients revealed a statistically highly significant association between IL-11 expression in primary tumors and an increased risk for development of bone metastasis^35^. The IL-11 protein levels in the serum and primary tumors were significantly higher in patients with bone metastasis compared to those without distant metastasis. Another similar study observed higher IL-11 transcript levels in breast cancer patients that relapsed 3–5 years after initial diagnosis when compared to a relapse-free cohort^36^. Although evidence has accumulated that IL-11 facilitates metastatic dissemination of cancer cells to distant sites^37^, IL-11 over-expression alone failed to increase the rate by which MDA-MB-231 cells formed bone metastases, suggesting that IL-11 may not be involved in the homing of disseminated cancer cells to bone^21^.

The activation of STAT3 is essential in IL-11 induced osteoclastogenesis. Studies also showed that excessive STAT3 activation is a feature of the majority of solid cancers in association with elevated IL-6 and IL-11 expression, including more than 40% of breast cancers^38,39^. In breast cancer cells, STAT3 activation is involved in a large number of phenotypic responses such as cell proliferation, survival, anti-apoptosis, angiogenesis and metastasis^39^. Phosphorylation inhibitors targeting STAT3 has already been proven effective in reducing tumor volume of pancreatic and breast cancers in mouse xenografts^40^. Our data suggested that p-STAT3 inhibition by AG-490 effectively reduced the osteolytic activity of BoM-1833 cells and the following release of TGF-β from bone matrix, thus inhibited the tumor growth. Considering the critical role STAT3 may play in the progression of breast cancer, the alleviated tumor growth in our study may also partially due to the direct inhibition of STAT3 activation in breast cancer cells. In each way, we regard STAT3 gains more significance as a therapeutic target in breast cancer bone metastases.

In conclusion, we demonstrated that bone metastatic breast cancer secreted IL-11 plays an essential role in the vicious osteolytic cycle by activating osteoclastogenesis independently of RANKL. IL-11 activated JAK1/STAT3 signaling further induces the expression of c-Myc, the necessary factor required for osteoclastogenesis. By inhibiting STAT3 phosphorylation, AG-490 is effective in reducing osteolysis and tumor growth of metastatic breast cancer, suggesting the potential of STAT3 as a therapeutic target in treating breast cancer bone metastases.

## Supplementary information

Supplemental methods and figures
