# Supplementary material for: IL-11 is essential in promoting osteolysis in breast cancer bone metastasis via RANKL-independent activation of osteoclastogenesis
Source: Cell Death Dis. 2019 Apr 30;10(5):353. doi: 10.1038/s41419-019-1594-1 (PMC6491651; doi:10.1038/s41419-019-1594-1)
Supplement: Supplementary file 1 — Supplemental methods and figures [file 41419_2019_1594_MOESM1_ESM.docx]

**IL-11 is Essential in Promoting Osteolysis in Breast Cancer Bone Metastasis via RANKL-independent Activation of Osteoclastogenesis**

Mengmeng Liang,^1^ Qinyu Ma,^2^ Ning Ding,^3^ Fei Luo,^2^ Yun Bai,^2^ Fei Kang,^1^ Xiaoshan Gong,^1^ Rui Dong,^1^ Jingjin Dai,^1^ Qijie Dai,^2^ Ce Dou,^2*^ and Shiwu Dong^1, 2, 4*^

^1^ Department of Biomedical Materials Science, Third Military Medical University, Chongqing 400038, China

^2^ Department of Orthopedics, Southwest Hospital, Third Military Medical University, Chongqing 400038, China.

^3^ Department of Blood Purification, General Hospital of Shenyang Military Area Command, Shenyang 110000, China

^4^ State Key Laboratory of Trauma, Burns and Combined Injury, Third Military Medical University, Chongqing 400038, China.

**Corresponding author:**

Shiwu Dong, Department of Biomedical Materials Science, School of Biomedical Engineering, Third Military Medical University, Gaotanyan Street No.30, Chongqing 400038, China. Telephone number: +86 2368771270. Email: [dongshiwu@tmmu.edu.cn](mailto:dongshiwu@tmmu.edu.cn)

Ce Dou, Department of Orthopedics, Southwest Hospital, Third Military Medical University, Gaotanyan Street No.30, Chongqing 400038, China. Email: lance.douce@gmail.com

**Supplementary Experimental Procedures**

**Reagents**

Anti-phospho-JAK1, anti-JAK1, anti-phospho-STAT3, anti-STAT3, anti-c-Myc, anti-c-Fos, anti-NFATc1, and anti-GAPDH were purchased from Santa Cruz Biotechnology(Dallas, TX, USA). STAT3 inhibitor AG-490 was obtained from MedChemExpress. Recombinant Mouse IL-11, recombinant Mouse RANKL and Recombinant Mouse M-CSF were purchased from R&D Systems (Minneapolis, MN, USA). TRAP stain kit was obtained from Sigma-Aldrich (NY, USA). Actin Cytoskeleton and Focal Adhesion Staining Kit was purchased from Millipore (Darmstadt, Germany). Monoclonal antibodies against VEGF, PTHrP, IL-11 and CTGF were purchased from Abcam (Cambridge, UA). Alpha minimal essential Medium (α-MEM) and fetal bovine serum (FBS) was purchased from Gibco (life technologies, NY, USA). Penicillin-streptomycin solution was obtained from Gibco (life technologies, NY, USA).

**Co-culture assay and CM preparation**

BMMs were cultured for 24 hours in the lower chambers of 24-well Transwell plates (Corning). Breast cancer cells (5×10^4^ cells) were seeded in the upper chamber and cocultured for an additional 72 hours. To confirm the level of osteoclastogenesis, the lower chamber cells were fixed in formalin and stained for TRAP. For conditioned medium (CM), cancer cells (1×10^5^ cells) were cultured in 100-mm dishes with 10% FBS-DMEM media. When cells were incubated till 80% confluent, cells were then washed with PBS and cultured with fresh DMEM for 24 hours. Then the CM was collected, filtered (0.2 μm) and kept at -80℃ for use.

**RT-qPCR**

Total RNA was isolated using Trizol reagent (Life Technologies, NY, USA). Single-stranded cDNA was prepared from 1 μg of total RNA using reverse transcriptase with oligo-dT primer according the manufacturer’s instructions (Promega, Madison, WI, USA). Two microlitres of each cDNA was subjected to PCR amplification using specific primers.

**Western blotting**

Cells were lysed in a lysis buffer containing 10 mMTris, pH 7.2, 150 mMNaCl, 5 mM EDTA, 0.1% SDS, 1% Triton X-100, and 1% deoxycholic acid. For Western blots, 30 μg of protein samples were subjected to SDS-PAGE followed by transfer onto PVDF membranes. After blocking in 5% skim milk, membranes were incubated with primary antibodies overnight at 4 °C. Primary antibodies used for immunoblotting include: anti-JAK1 (Santa Cruz) at a 1:1000 dilution, anti-p-JAK1 (Santa Cruz) at a 1:1000 dilution, anti-STAT3 (Santa Cruz) at a 1:500 dilution, anti-p-STAT3 (Santa Cruz) at a 1:500 dilution, anti-c-Myc (Santa Cruz) at a 1:1000 dilution, anti-c-Fos (Santa Cruz) at a 1:1000 dilution, anti-NFATc1 (Santa Cruz) at a 1:1000 dilution, anti-GAPDH (Santa Cruz) at a 1:5000 dilution. Rabbit antibodies against primary antibodies followed by 1 h-incubation with secondary antibody (1:2000). Blots against GAPDH served as loading control. Chemiluminescent signals were detected using Immun-Star HRP (BioRad).

**Supplementary Figures**


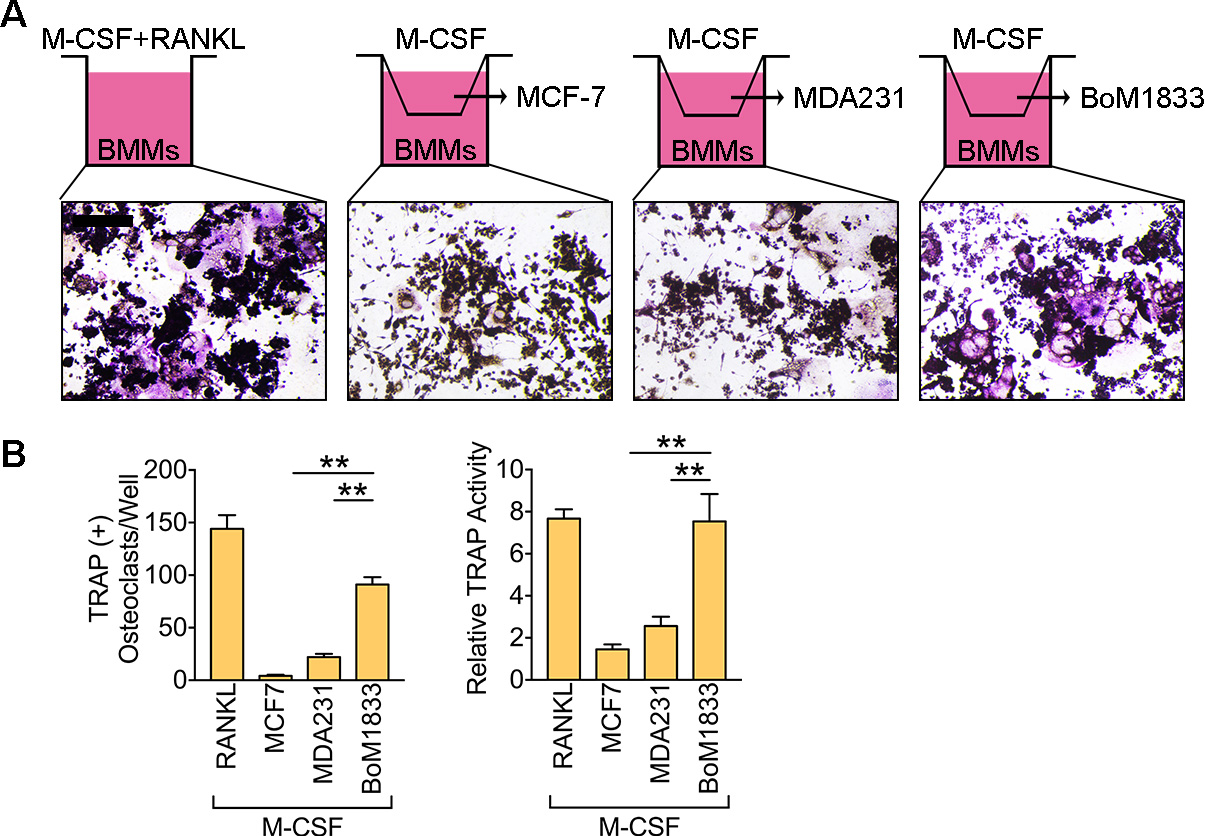


**Supplementary Fig. 1** (A) Representative TRAP stain images of BMMs treated with M-CSF+RANKL, or cocultured with MCF-7, MDA-MB-231 or BoM-1833 cells with M-CSF treatment. Bar represents 200 μm. (B) Quantification of relative TRAP activity and osteoclast number per well. The data in the figures represent the averages ± SD. Significant differences are indicated as * (*p* < 0.05) or ** (*p* < 0.01) paired using Student’s t test unless otherwise specified.


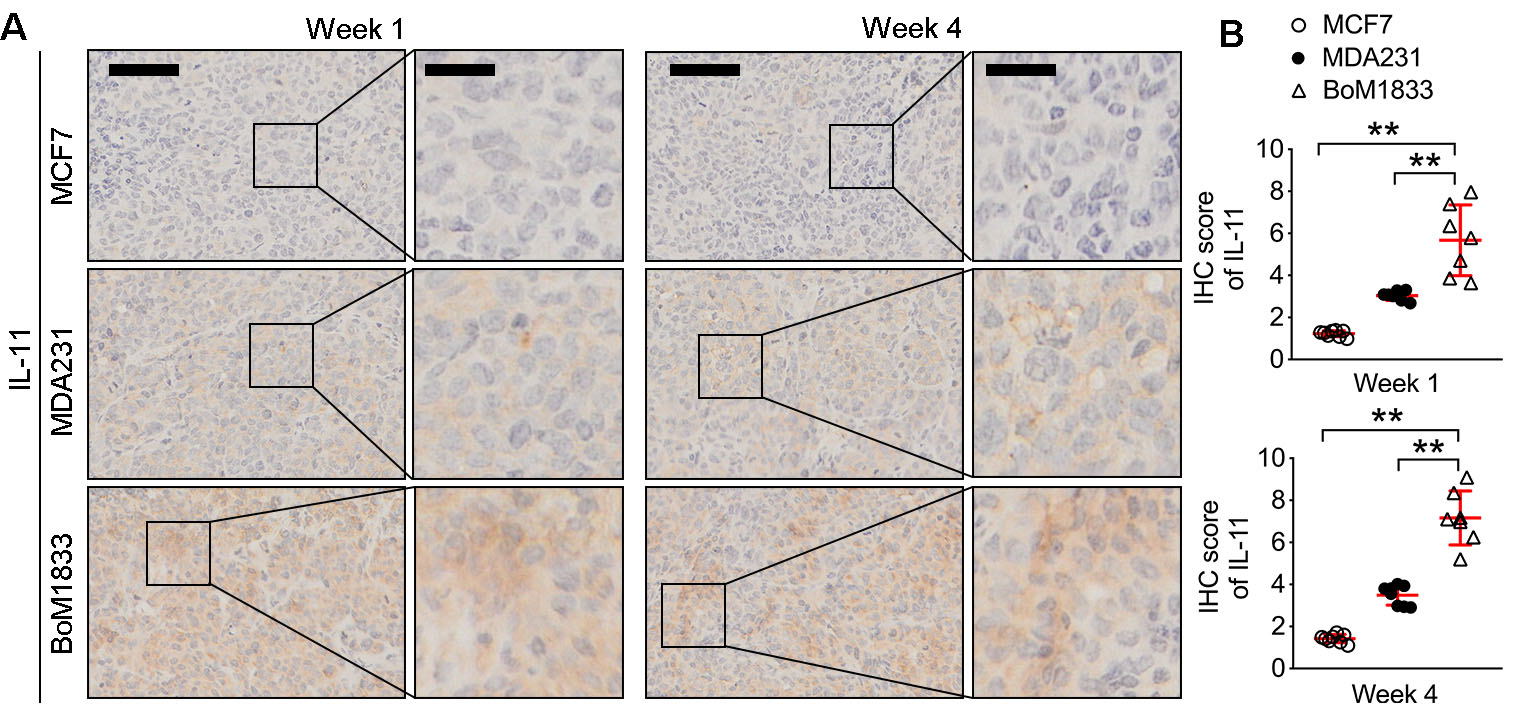


**Supplementary Fig. 2:** (A) Representative immunohistochemical (IHC) stain images of IL-11 of distal femurs in week 1 and week 4 of mice with intrafemoral injection of MCF7, MDA-MB-231 or BoM-1833 cells. Bar represents 200 μm (left) and 50 μm (right). (B) IHC scores of IL-11 were quantified. The data in the figures represent the averages ± SD. Significant differences are indicated as * (*p* < 0.05) or ** (*p* < 0.01) paired using Student’s t test unless otherwise specified.


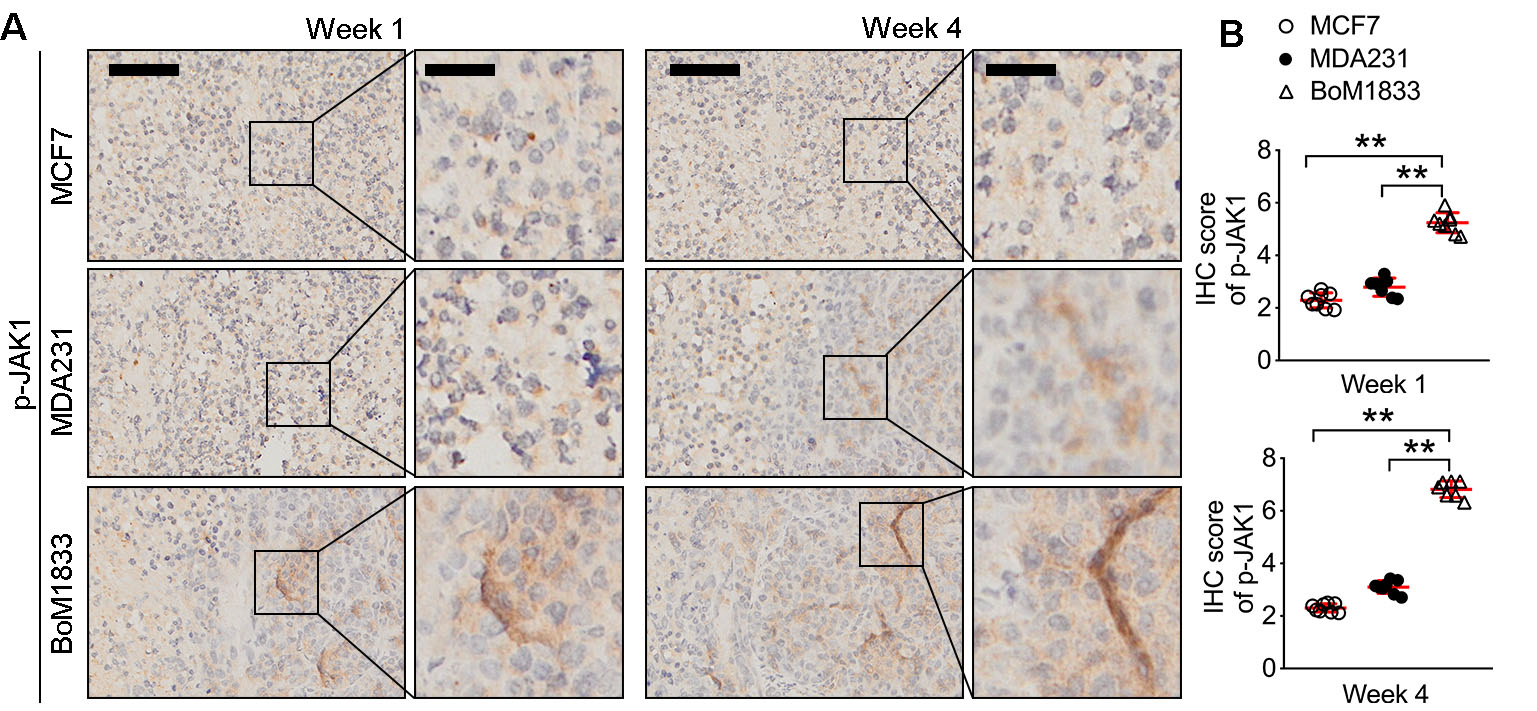


**Supplementary Fig. 3:** (A) Representative immunohistochemical (IHC) stain images of p-JAK1 of distal femurs in week 1 and week 4 of mice with intrafemoral injection of MCF7, MDA-MB-231 or BoM-1833 cells. Bar represents 200 μm (left) and 50 μm (right). (B) IHC scores of p-JAK1 were quantified. The data in the figures represent the averages ± SD. Significant differences are indicated as * (*p* < 0.05) or ** (*p* < 0.01) paired using Student’s t test unless otherwise specified.


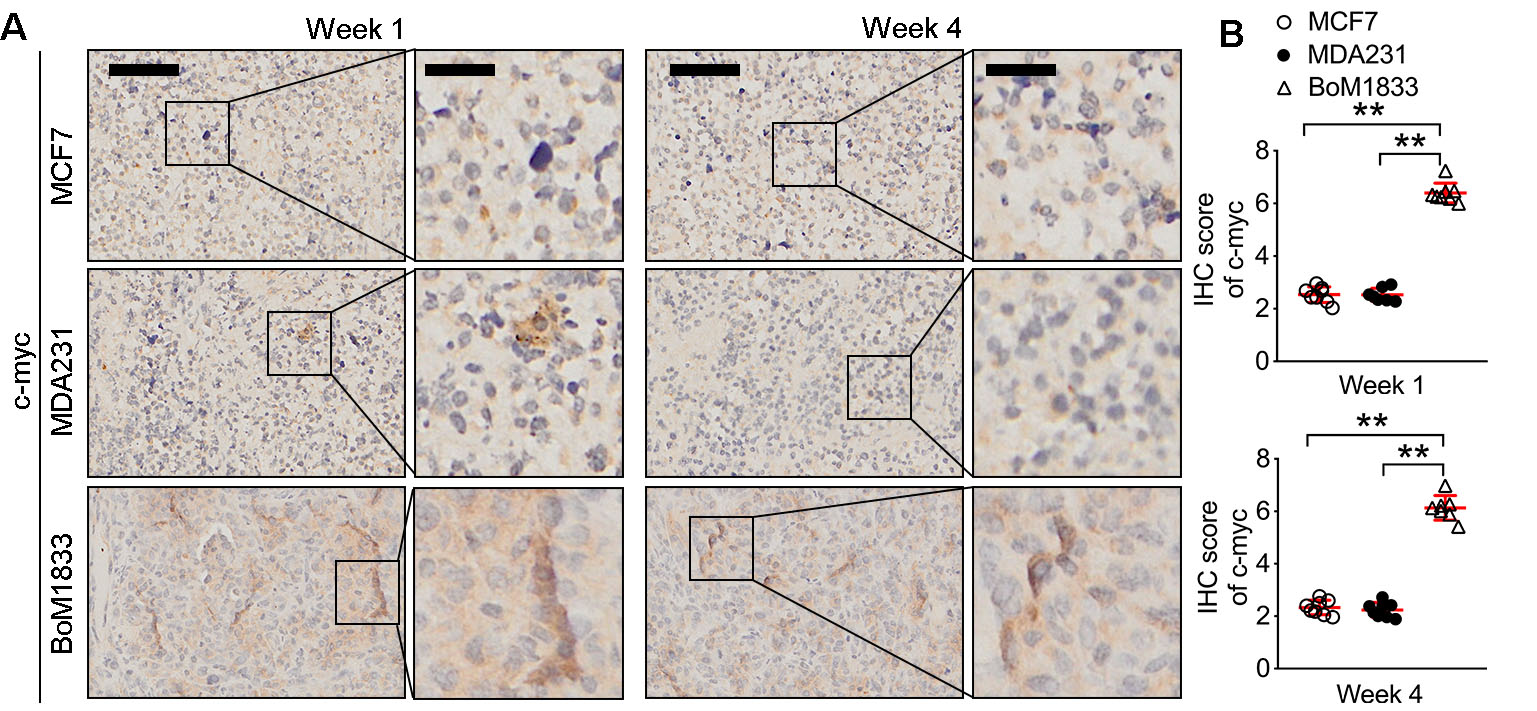


**Supplementary Fig. 4:** (A) Representative immunohistochemical (IHC) stain images of c-myc of distal femurs in week 1 and week 4 of mice with intrafemoral injection of MCF7, MDA-MB-231 or BoM-1833 cells. Bar represents 200 μm (left) and 50 μm (right). (B) IHC scores of c-myc were quantified. The data in the figures represent the averages ± SD. Significant differences are indicated as * (*p* < 0.05) or ** (*p* < 0.01) paired using Student’s t test unless otherwise specified.


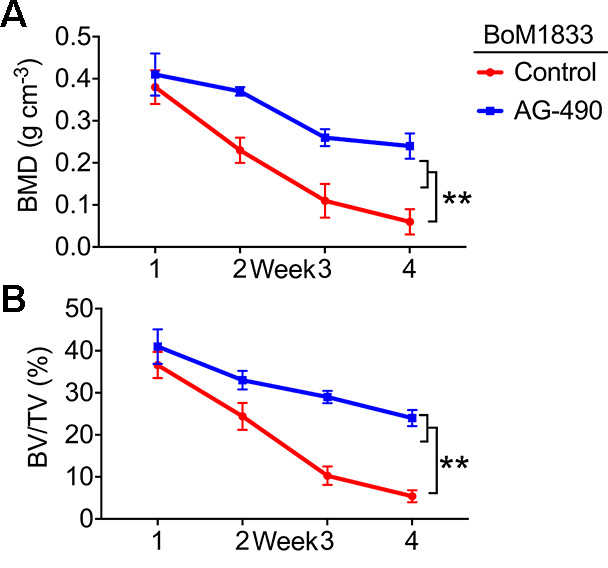


**Supplementary Fig. 5** Quantification of bone mineral density (BMD) (**A**) and trabecular bone volume fraction (BV/TV) (B). The data in the figures represent the averages ± SD. Significant differences are indicated as * (*p* < 0.05) or ** (*p* < 0.01) paired using Student’s t test unless otherwise specified.


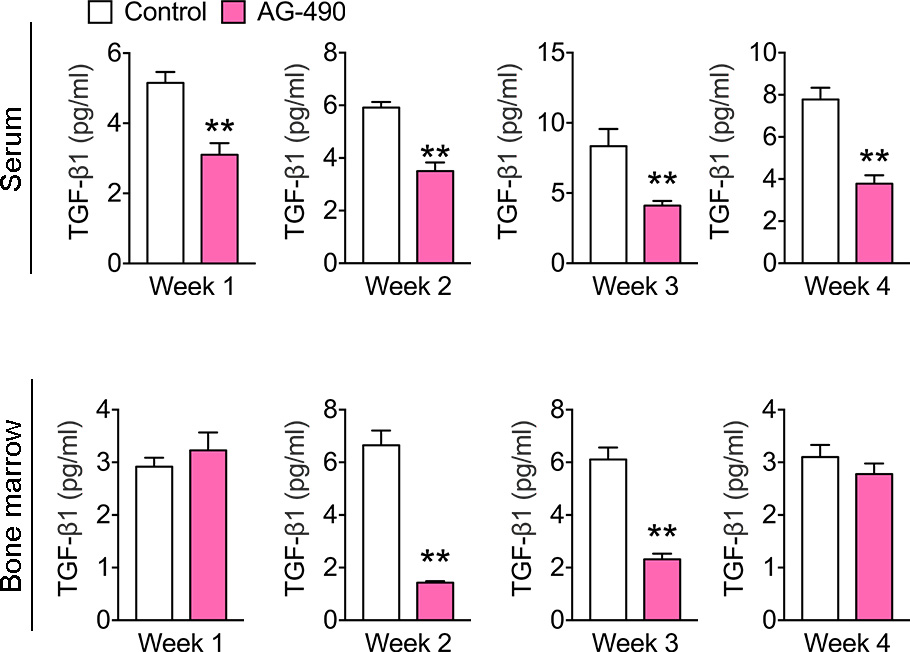


**Supplementary Fig. 6:** ELISA for serum or bone marrow (BM) TGF-β concentrations. The data in the figures represent the averages ± SD. Significant differences are indicated as * (*p* < 0.05) or ** (*p* < 0.01) paired using Student’s t test unless otherwise specified.


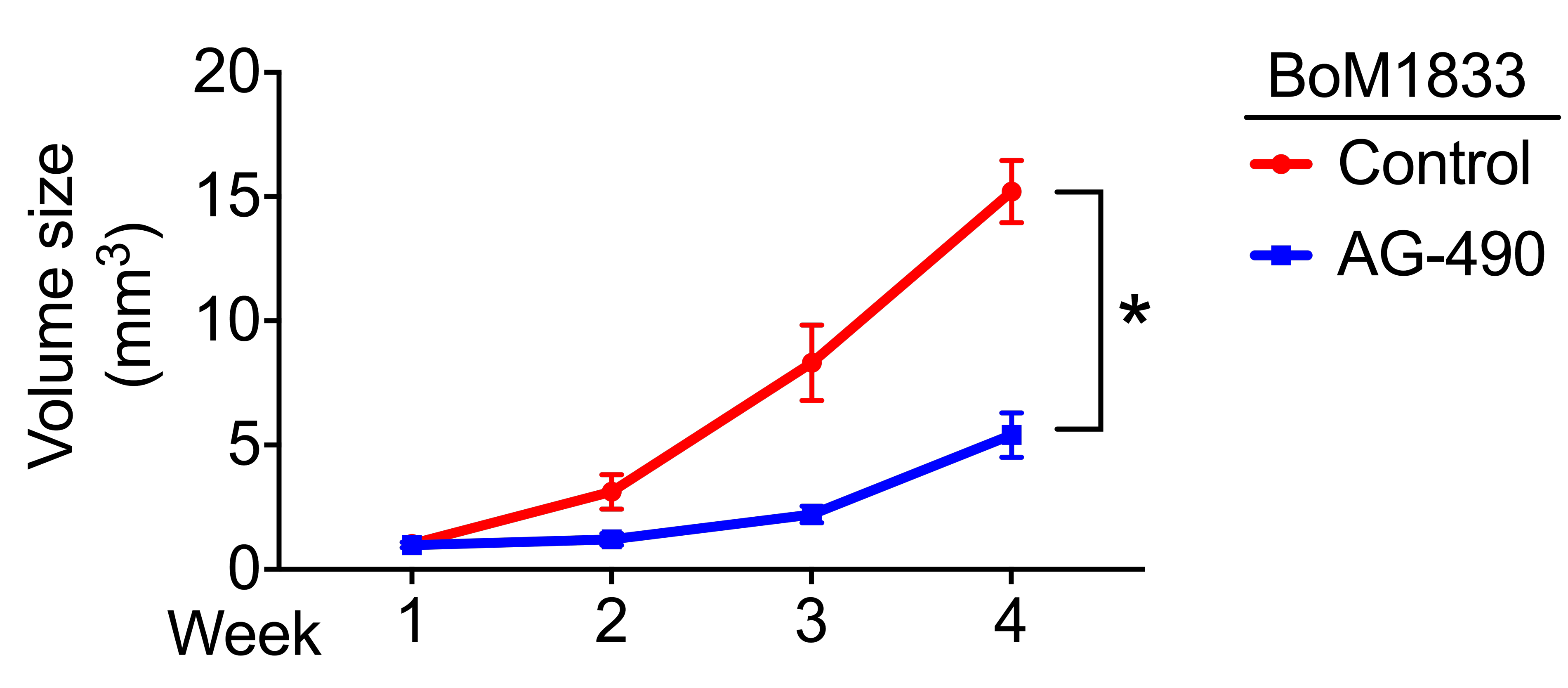


**Supplementary Fig. 7:** Quantification of normalized tumor volume size. The data in the figures represent the averages ± SD. Significant differences are indicated as * (*p* < 0.05) or ** (*p* < 0.01) paired using Student’s t test unless otherwise specified.
